# Annotations

**Published:** 1900-03-31

**Authors:** 


### Annotations.

### Paris as a School of Manners.

All sorts of people no doubt intend to go to the Paris Exhibition, and among them will certainly be a considerable assignment of those ill-regulated and dirty folk whom we may call "spitters." Let them, then, begin at once and put themselves in training, for spitting is to be prohibited in Paris! Still, notwithstanding what we hear about the determination of the Municipal Council in Paris to put a stop to spitting in the streets of that gay city, we greatly question whether the London County Council, or any of the minor bodies charged with upholding decency in our midst will follow suit. When electricity shall hold the field, and horse dung and other filth shall have ceased to give to London air that peculiar fragrance by which it is distinguished, then perhaps it will be possible to prohibit spitting in the streets, but at present a sense of humour and of due proportion in evil doing will probably hold us back from any extreme measures. In the meantime, we might, perhaps, go so far as is done in some American cities; that is, prohibit expectoration on the footpaths or, as they call them, the "sidewalks." This would be no hardship. We doubt, however, whether even this minor step will be taken. A generation so callous and indifferent to decency that great public omnibus companies can allow their drivers to while away the time by repeated and apparently aimless spitting in free air to the annoyance of their own customers, cannot feel very acutely on the subject; and, indeed, we feel rather inclined to join with Lady Harberton, who points out that even the ladies, whom we always associate with everything delicate and nice, show but little sign of sensitiveness in this nasty subject, or they would not walk about in trailing skirts. With such indifference on every side, much as we may grumble and dirty as the habit is, we fear that it will persist. Still, if a desire to go to Paris should lead a few "spitters" to train themselves in better ways, we shall certainly wish every success and many visitors to the exhibition.

### Antivivisection.

The Metropolitan Radical Federation has chosen to intervene in the antivivisection question, and has drawn up a memorial which if taken seriously must do harm to the hospitals; but Mr. Melhado, secretary superintendent of the Middlesex Hospital, in a letter to the *Times* states that this so-called memorial is practically a reproduction of an article by the Hon. Stephen Coleridge which recently appeared in the *Contemporary Review*. So are memorials manufactured, and "Radical" and other "Federations" made to dance to the tune of the pipers in Victoria Street. It is time there was some common sense let into this weary talk about vivisection. It ought to be understood by the public that the hospitals are being attacked because they are trying to do their work well, and because certain medical men attached to them, or to their associated medical schools, are willing to put their feelings on one side, and to do things which they often find loathsome and repulsive, namely to do certain operations upon animals, in order that progress in knowledge may be made, that disease may be conquered, that suffering may be prevented, and that human life may be prolonged. If the hospitals would go on in the "good old ways," would drench their unfortunate patients with gallons of useless physic, and would cut legs off instead of saving them, then we suppose these antivivisectors would be content. But because, in addition to the countless amount of money given for the maintenance of these great charities, and in addition to the sickness borne, and the lives laid down by doctors and nurses alike in the service of their patients, the lives of a few rabbits also are to be sacrificed for the benefit of humanity—then there is an outcry. England wants to be brought again face to face with the hard facts of life, and then this maudlin sentimentality among the idle members of the population would dissolve away. We would be the last to advocate cruelty to animals, but there should be some sense of proportion in all things. At a time when we are condemning thousands of our fellow men to the horrible tortures involved by war, and consider ourselves excused in so doing by the great necessity, so in view of the sufferings of women and children we hold ourselves justified even in pricking a rabbit should there seem any likelihood of giving them relief.

### The Woes of the Profession.

However successful some amongst us may be in regard to this world's goods we hear on every side that as a profession we are the victims of much injustice, and that our services are neither appreciated nor paid for as they ought to be. Now according to our temperament and frame of mind will be the cure which we are likely to propound for this distressing and very improper state of affairs. There are some of us who, resting our faith upon the inherent justice of our cause, and innocently believing that the General Medical Council, being paid for by our fees, must have been created by a kind providence for our protection, would throw upon that body the pleasing duty of excommunicating all doctors who presume to accept a rate of remuneration lower than seems appropriate to our position and our dignity, while there are others who are sufficiently men of the world to see that if the medical profession wishes to raise its remuneration it must do as every other trade or profession does under like circumstances, that is, it must combine for the purpose. And why should we not combine? Combination ought to be even more successful in regard to the remuneration for medical services than is the case in regard to other forms of "labour," for the medical profession is a close corporation into the fold of which no outsiders can intrude without going through a long and expensive training. Hence, we ought to have the ball at our feet if we would but combine. But we do not, even for so urgent an object as that of fixing such a minimum as a penny a week (!) for our remuneration. Correspondents on every side keep pouring out their woes upon the readers of the medical papers, and we are truly sorry for those practitioners who have to put up with the treatment to which they seem to be exposed. When we read these professional woes we all must feel that surely something ought to be done to remove the hardships there displayed. But take the profession in the lump we stand impassive. We must be a flinty-hearted lot to let our brethren starve rather than take the simple measure of combining for mutual protection—or else the cry is not quite so real as some would have it.

